# The Moderating Effect of Labor Force Participation on the Prevalence of Depression Among Older Adults

**DOI:** 10.1093/geroni/igaf122.444

**Published:** 2025-12-31

**Authors:** Antonia Diaz-Valdes, Jose Medina, Esteban Calvo, Nicolas Montalva

**Affiliations:** Universidad Mayor, Santiago, Region Metropolitana, Chile; Universidad Mayor, Santiago, Region Metropolitana, Chile; Universidad Mayor, Santiago, Region Metropolitana, Chile; Universidad Mayor, Santiago, Region Metropolitana, Chile

## Abstract

Depression among older adults is one of the most pressing issues in public health, yetis one of the less recognized problems and is one of the leading causes of disability. This study aims to examine cross-national gender gaps in the prevalence of depression risk from 2000 to 2020, controlling for age and cohort effects, and exploring labor force status as moderator. A random-effects model with robust standard errors was conducted to examine depression cases and predict probabilities. Our analysis involved a sample of 246,000 individuals aged 50 and older from 35 countries with at least 2 waves of reported measures, spanning the years 2000-2020. We used cut-points for risk of depression based on the depression scales used in the different surveys – Health and Retirement Study and sister datasets around the world. The average predicted probability of depression was 22.00 % for males and 33.62% for females. While the gender gap in depression probability remained stable over time, both males and females exhibited an upward trend in depression probability during this period – about 3.52 and 3.79 respectively. The average predicted probability of depression varied by labor status, being 19.32% and 31.70% for working males and females respectively, and 23.00% for retired males and 34.27% for retired females. Our study highlights the importance of screening and treating depression among older adults to reduce the consequences of depression, especially among women, who are disproportionately affected independent of their labor force status

